# Leptin receptor (LEPR) SNP polymorphisms in HELLP syndrome patients determined by quantitative real-time PCR and melting curve analysis

**DOI:** 10.1186/1471-2350-11-25

**Published:** 2010-02-11

**Authors:** Tibor Várkonyi, Levente Lázár, Attila Molvarec, Nándor Gábor Than, János Rigó, Bálint Nagy

**Affiliations:** 1 1st Department of Obstetrics and Gynecology, Semmelweis University, 1088 Budapest, Hungary; 2 Perinatology Research Branch, Eunice Kennedy Shriver National Institute of Child Health and Human Development, National Institutes of Health, Department of Health and Human Services, Detroit, MI 48201, USA

## Abstract

**Background:**

Several studies have shown overexpression of leptin in microarray experiments in pre-eclampsia (PE) and in hemolysis, elevated liver enzymes, low platelets (HELLP) syndrome. We decided to study four leptin receptor (*LEPR*) SNP polymorphisms in HELLP syndrome patients by using quantitative real-time PCR and melting curve analysis.

**Methods:**

DNA was isolated from blood samples from 83 normotensive pregnant women and 75 HELLP syndrome patients. Four SNPs, *LEPR c.326A>G *(K109), *LEPR c.668A>G *(Q223R), *LEPR c.1968G>C *(K656N) and *LEPR c.3024A>G *(S1008) were determined by quantitative real-time PCR and melting curve analysis. Investigators were blinded to clinical outcomes.

**Results:**

*LEPR c.326A>G*, *LEPR c.668A>G*, *LEPR c.1968G>C *and *LEPR c.3024A>G *allele, genotype and haplotype polymorphisms were not different in HELLP syndrome patients and normotensive healthy pregnants. There were strong linkage disequilibrium (LD) between loci *c.326A>G *and *c.6687A>G *(D' = 0.974), and *c.668A>G *and *c.1968G>C *(D' = 0.934), and *c.326A>G *and *c.1968G>C *(D' = 0.885), and *c.1968G>C *and *c.3024A>G *(D' = 1.0). However, linkages of *c.3024A>G *with *c.668A>G *(D' = 0.111) and *c.326A>G *(D' = 0.398) were weak. The Hardy-Weinberg equilibrium was observed for all polymorphisms. However the *LEPR c.326A>G AG *genotype was twice more frequent and the (AG AG GG AG) haplotype was three times more frequent in HELLP syndrome patients. The introduced quantitative real-time PCR combined with melting curve analysis is a fast and reliable method for the determination of *LEPR *SNPs.

**Conclusion:**

Although certain *LEPR *haplotypes are more frequent in HELLP syndrome, we conclude that there is no compelling evidence that the four studied *LEPR *SNP polymorphisms associated with the development of HELLP syndrome.

## Background

The human leptin receptor is a member of class 1 cytokine receptor family, which was identified in the brain [1]. The gene was assigned to chromosome 1. There are six isoforms of the leptin receptor. The longest is the most abundant in the hypothalamus, which is responsible for leptin signaling. The shortest isoform is predominant in peripheral tissues, and there is a soluble isoform as well [2-6]. Leptin acts through the leptin receptor (*LEPR*). Leptin circulates in free and in a leptin receptor bound form. It acts via the receptors located in adipose tissue, stomach, endometrium, liver, spleen, lungs, heart, ovaries and placenta. Binding to the soluble receptor might influence the amount of biologically available ligand [2,7-9].

Leptin regulates the food intake and energy metabolism, it was found that beside that, it stimulates the proliferation of various cell types and it is considered to be a new growth factor. It seems to have effect on immunity, angiogenesis, reproduction, and in the regulation of the blood pressure [10,11]. Leptin is a mitogenic as well as pro-angiogenic factor in various cells [12]. Leptin acts synergistically with VEGF (vascular endothelial growth factor) and fibroblast growth factor 2 (FGF-2) to promote angiogenesis [13]. It has effect on the expression of several genes involved in the angiogenesis (MMP-2 and MMP-9) [14,15]. Leptin was implicated in the pathogenesis of several disease including essential hypertension and pre-eclampsia (PE) [16,17].

There are several studies showing higher expression of the leptin gene in microarray experiments in PE and HELLP syndrome [18-21]. As there are polymorphisms which have effect on gene expression [22], and only one study was dealing with two LEPR SNPs in PE [10]. However, the *LEPR *polymorphism has not been studied in HELLP syndrome (hemolysis, elevated liver enzymes, and low, platelet count), in the disease which is considered as a severe complication of PE. We decided to determine the LEPR SNP polymorphism in HELLP syndrome and normal healthy subjects.

The determination of *LEPR *SNPs is mostly based on PCR-RFLP, Mass Array genotyping and TaqMan determinations in previous studies [22-26]. We introduced first time the quantitative real-time PCR and melting curve analysis method to determine those SNPs which seemed to have functional relevance and suspected to effect circulating leptin levels [10].

## Methods

Between January 2003 and September 2008, 75 consecutive Hungarian HELLP syndrome patients and 83 normotensive healthy pregnant women were enrolled and tested in the study for the leptin receptor SNP polymorphism at the 1^st ^Department of Obsterics and Gynecology at the Semmelweis University Budapest, Hungary. We determined the four SNPs in all samples. The mean characteristics of the groups are shown in Table 1. The healthy control patients were consecutively selected from a group of normotensive, healthy pregnant women, who were undergoing routine blood tests, and were excluded if they developed hypertensive disorder.

**Table 1:** Clinical characteristics of the patients involved in the study

	Normotensive healthy pregnants	HELLP syndrome patients	p value*
	n = 83	n = 75	
Average age (years)	31.76 ± 3.93	30.08 ± 5.12	ns
BMI before pregnancy (kg/m^2^)	23.29 ± 3.88	25.99 ± 4.29	ns
Primipara	33 (40%)	43 (57%)	ns^§^
Systolic blood pressure (Hgmm)	121.8 ± 11.2	155.4 ± 19.4	0.0001
Diastolic blood pressure (Hgmm)	77.3 ± 7.9	97.8 ± 12.8	0.0001
Gestational age at delivery (week)	39.63 ± 1.87	31.2 ± 3.72	0.0001
Newborn's weight (gramm)	3504.76 ± 426.2	1431.38 ± 700	0.0001
IUGR	0%	30 (40%)	0.0001
Smoking	6 (7%)	5 (7%)	ns

A *p *value was calculated using the *Student's *t*-test or ^†^Chi-Squared test.

Significant value was taken at the level of *p *< 0.05.

ns = non-significant.

In the case of HELLP syndrome cases the following criteria were defined: hemolysis, classified by increased lactic dehydrogenase activity (>600 IU/L); elevated liver enzyme activity, defined as increased aspartate aminotransferase and alanine aminotransferase levels (> 70 IU/L); and thrombocytopenia (≤ 100 platelets × 10^9^/L) [27]. IUGR was diagnosed in neonates when their birth weight was below 10 percentile for gestational age by weight based on Hungarian population tables.

The Ethical Committee of the Semmelweis University has approved the study, all participants were informed and they agreed in their involvement in the study and they signed consent.

Three ml of peripheral blood was drawn from each patient into an EDTA tube. Genomic DNA was extracted from 0.2 ml samples by using the High Pure PCR Template Isolation kit (Roche, Mannheim, Germany), according to the manufacturer's instructions [28,29].

Four *LEPR *SNPs were determined by quantitative real-time PCR and melting curve analyses on the LightCycler instrument (Roche GmbH, Penzberg, Germany) [rs1137100 (*G+5193A; c.326A>G*); rs1137101 (*G+27265A; c.668A>G*); rs8179183 (*G+44704C; c.1968G>C*); rs6413506 (*A+71001G; c.3024A>G*)]. Table 2 shows the primer and probe sequencies, they were produced and designed by TibMolbiol (Berlin, Germany). The PCR mix contained 1 μl of genomic DNA, 5 μM primers and probes, 1 μl of LightCycler FastStart DNA Master HybProbe solution (Roche), and 4.5-5.0 mM MgCl_2_. PCR condition and melting curve reading parameters were optimized. The annealing temperature was 52-62°C.

**Table 2:** LEPR SNP primer and probe sequencies

**LEPR c.326A>G**	
Forward	5'-AgTggTACTCACTTTTCTAACTTATC-3'
Reverse	5'-gAATTAAAAAACATTgTTCAATACA-3'
Sensor	5'-AACATTgAAggAA(A/g)gACATTTgTT-Fluorescein
Anchor	LC640-CAACAgTAAATTCTTTAgTTTTTCAACAAATAgg-Phosphate
	
**LEPR c.668A>G**	
Forward	5'-CAgCCAAACTCAACgACACT-3'
Reverse	5'-CCACTCTTAATACCCCCAgTACTA-3'
Sensor	5'-CATTAgAggTgAC(T/C)ggAAAATTAC-Fluorescein
Anchor	LC640-CCACCAgATgTgATTTTCAAACACATAAgg-Phosphate
	
**LEPR c.1968G>C**	
Forward	5'-CAACTTgTCATTTTgCAgTTCCTA-3'
Reverse	5'-gCTTTCCgAAgATTAATAACAggAT-3'
Sensor	5'-TgACATTTTTCTC(C/g)TTTTTCATAgTATC-Fluorescein
Anchor	LC640-CCATTAATTATTCTCCAAAATTCAggTCCT-Phosphate
	
**LEPR c.3024A>G**	
Forward	5'-CCAgAgACAACCCTTTgTTAAATACg-3'
Reverse	5'-TggTgAAATTATgTTgggATgC-3'
Sensor	5'-gCACTTggTgAC(T/C)gAACTATTTAT-Fluorescein
Anchor	LC640-gCCCTTgTTCTTCACCAgTTTCACTT-Phosphate

The wild-type and the variant sequences showed different melting peaks, representing a distinguishable melting point (T_m_), while heterozygotes had both peaks during the melting curve analysis.

**Figure 1:**
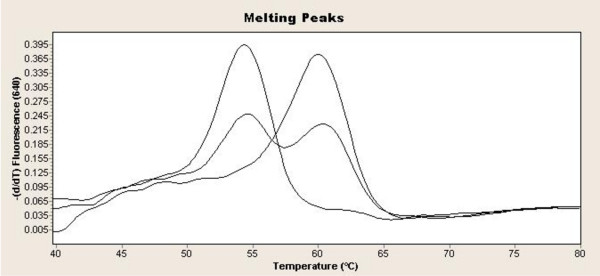
**Melting curve analysis of the *LEPR G+5193A***. The figure shows the melting curves of the *LEPR G+5193A *in wild type, a variant and heterozygote sample. The melting point (T_m_) of the wild type is 61°C and 55°C for the variant, the heterozygote sample has both peaks.

The *LEPR c.326A>G *has the T_m _63°C for wild type and 55°C for the variant (Figure 1). The *LEPR c.668A>G *has the T_m _63°C for the wild type and 55°C for the variant. The *LEPR c.1968G>C *has the T_m _63°C for the wild type and 57°C for the variant. The *LEPR c.3024A>G *has the T_m _47°C and 54°C for the variant.

Sample size estimation of the study was performed by using Quanto 1.2.4 http://www.hydra.usc.edu statistical program. Abate et al [30] published their data on Lys109Arg LEPR polymorphism with a similar set up. Our sample size provided sufficient statistical power (>80% at Type I error rate of 0.05) to detect 21% difference in LEPR c.326A>G genotypes and 14% difference in LEPR (AG AG GG AG) haplotypes between cases and controls [31].

Statistical analyses were performed with the STATISTICA software package (version 8; StatSoft, Inc., Tulsa, Oklahoma, USA). The statistical significance of the differences between patient groups was evaluated by Mann-Whitney non-parametric U-test p < 0.05 was considered as statistically significant.

Pearson Chi-square (χ^2^) test was used for comparing groups of categorical data (allele, genotype frequencies). The haplotype analysis and control for deviation from the Hardy-Weinberg equilibrium for the studied *LEPR *polymorphisms was conducted using Haploview software http://www.broad.mit.edu/mpg/haploview. Haplotype frequencies were estimated with linkage disequilibrium coefficient.

## Results

PCR parameters were optimized for the four primer-probe system to obtain easily recognizable T_m _differences among the different alleles for detection of *LEPR *SNPs (Figure 1). DNA samples of 83 healthy pregnant and 75 HELLP syndrome patients were studied. There was no statistically significant difference in the average maternal age, the percentage of smokers and primiparas between the HELLP and healthy pregnant control group (Table 1).

Table 3 shows the allele distribution of the four SNPs. There was no statistically significant difference in the distribution of the alleles in the studied groups. The frequency of the *A *allele was 77.0% in the HELLP syndrome group and 73.0% in the healthy pregnant controls in the case of *LEPR c.326A>G *(K109). Our sample size provided sufficient statistical power (>80% at Type I error rate of 0.05) to detect 21% difference in LEPR c.326A>G genotypes. The *LEPR c.668A>G *(Q223R) showed 55.0% of the *A *allele in HELLP cases compared to 58.0%. The frequency of the *G *allele was 80.0% in the HELLP patients and 84.0% in the healthy controls in the case of *LEPR c.668A>G *(K656N). The frequency of the *A *allele was 38.0% and 32.0% in the *LEPR c.3024A>G *(S1008).

**Table 3:** Allele and genotype frequencies for the four polymorphisms of the LEPR gene

	Allele^a^					Genotype			
	
	n	1^b^	2^b^	χ^2 ^(P value)	Odds ratio (95% CI)	11^b^	12^b^	22^b^	χ^2 ^(P value)
**LEPR c.326A>G**									
Control	83	38 (23%)	128 (77%)	0.6 (p = 0.437)	0.81 (0.49-1.36)	9 (11%)	20 (24%)	54 (65%)	4.83 (p = 0.089)
HELLP	75	40 (17%)	110 (73%)			5 (7%)	30 (40%)	40 (53%)	
**LEPR c.668A>G**									
Control	83	69 (42%)	97 (58%)	0.3 (p = 0.578)	0.88 (0.56-1.38)	18 (22%)	33 (40%)	32(38%)	0.48 (p = 0.785)
HELLP	75	67 (45%)	83 (55%)			17 (23%)	33 (44%)	25 (33%)	
**LEPR c.1968G>C**									
Control	83	140(84%)	26 (16%)	1.01 (p = 0.313)	0.74 (0.41-1.32)	59 (71%)	22 (27%)	2 (2%)	1.01 (p = 0.602)
HELLP	75	120 (80%)	30 (20%)			48(64%)	24 (32%)	3 (4%)	
**LEPR c.3024A>G**									
Control	83	113 (68%)	53 (32%)	1.28 (p = 0.258)	0.76 (0.48-1.2)	10 (12%)	33 (40%)	40 (48%)	3.66 (p = 0.16)
HELLP	75	93 (62%)	57 (38%)			8 (11%)	41 (54%)	26 (35%)	

^a ^Note. Allele 1 and 2 represent the first and second nucleotides given in the name of the SNP, respectively

^b ^n(%)

Table 3 shows the genotype frequencies of the four SNPs. There was no significant difference in the distribution of the genotypes in the study groups. However a difference was observed in the frequency of the *LEPR c.326A>G AG *genotype, which was almost twice as frequent in HELLP syndrome, while it was not statistically significant (40.0% vs. 24.0%; p = 0.089).

Table 4 shows the haplotype frequencies of the SNPs in the study groups. We identified 24 haplotypes, however the (AG AG GG AG) was three times more frequent in HELLP syndrome (4 vs. 12).

**Table 4:** Haplotype combination frequencies of the examined four polymorphisms in LEPR gene in HELLP patients and normotensive healthy pregnant controls.

Haplotype combination				Control		HELLP	
LEPR c.326A>G (K109)	LEPR c.668A>G (Q223R)	LEPR c.1968G>C (K656N)	LEPR c.3024A>G (S1008)	n	frequencies	n	frequencies
AA	AA	GG	GG	16	0,19	10	0,13
AA	AA	CG	AG	12	0,14	10	0,13
AA	AG	GG	GG	12	0,14	6	0,08
AA	AG	CG	AG	5	0,06	7	0,09
AG	AG	GG	GG	5	0,06	3	0,04
AG	AG	GG	AG	4	0,05	12	0,16
GG	GG	GG	AA	4	0,05	2	0,03
GG	GG	GG	AG	4	0,05	0	0,00
AG	GG	GG	AG	3	0,04	5	0,07
AA	GG	GG	GG	3	0,04	2	0,03
AG	GG	GG	GG	2	0,02	4	0,05
AA	AA	CC	AA	2	0,02	3	0,04
AG	AG	CG	AG	2	0,02	3	0,04
AG	AG	CG	AA	2	0,02	2	0,03
GG	GG	GG	GG	1	0,01	2	0,03
AA	AA	GG	AG	1	0,01	1	0,01
AA	GG	GG	AG	1	0,01	0	0,00
AA	AG	GG	AG	1	0,01	0	0,00
AA	AG	CG	AA	1	0,01	0	0,00
AG	AA	GG	GG	1	0,01	0	0,00
AG	AG	GG	AA	1	0,01	0	0,00
AA	AA	CG	AA	0	0,00	1	0,01
AG	GG	GG	AA	0	0,00	1	0,01
GG	GG	CG	AG	0	0,00	1	0,01

The Hardy-Weinberg equilibrium was observed for all polymorphisms (Table 5).

**Table 5:** The genotype frequencies of LEPR polymorphism

		Allele frequency		HW
LEPR SNP	% tested	Major	Minor	P-value
c. 326 A>G	100	A: 0.753	G: 0.247	0.06127
c. 668 A>G	100	A: 0.57	G: 0.43	0.06276
c. 1968 G>C	100	G: 0.823	C: 0.177	0.82495
c. 3024 A>G	100	G: 0.652	A: 0.348	0.68796

HW = Hardy-Weinberg equilibrium

lag out condition: 0.5 < Sample Missing, 0.1 < SNP Missing, 0.05 > SNP MAF, 0.05/4 = 0.0125 > SNP HWE p-value, with multiple correction

**Figure 2:**
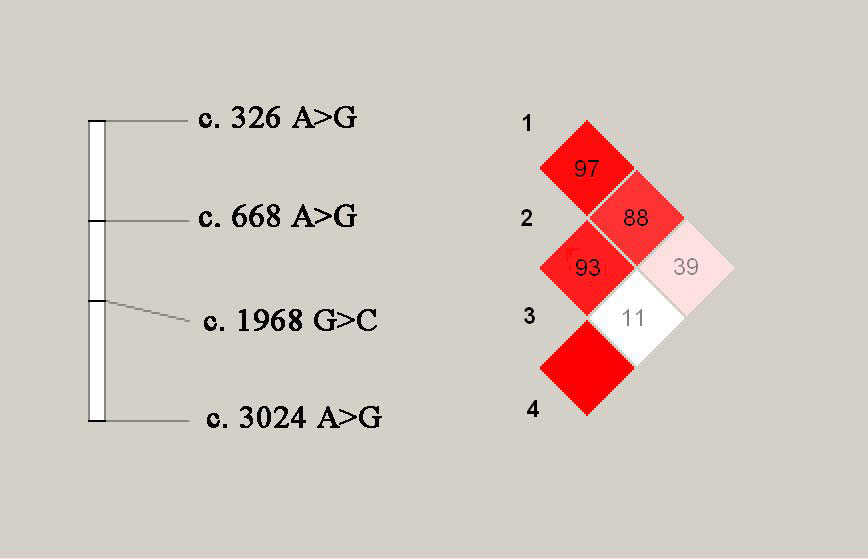
**Haplotype analysis of the four studied SNPs**. There were strong linkage disequilibrium (LD) between loci *c.326A>G *and *c.6687A>G *(D' = 0.974), and *c.668A>G *and *c.1968G>C *(D' = 0.934), and *c.326A>G *and *c.1968G>C *(D' = 0.885), and *c.1968G>C *and *c.3024A>G *(D' = 1.0).

The LDs of LEPR polymorphism at loci at *c.326A>G*/c.668A>G/*c.1968G>C*/*c.3024A>G *are shown in Figure 2. There were strong linkage disequilibrium (LD) between loci *c.326A>G *and *c.668A>G *(D' = 0.974), and *c.668A>G *and *c.1968G>C *(D' = 0.934), and *c.326A>G *and *c.1968G>C *(D' = 0.885), and *c.1968G>C *and *c.3024A>G *(D' = 1.0). However, linkages of *c.3024A>G *with *c.668A>G *(D' = 0.111) and *c.326A>G *(D' = 0.398) were weak.

## Discussion

We determined four coding polymorphisms in *LEPR *gene in HELLP syndrome patient by using quantitative real-time PCR and melting curve analysis method, first time according to our knowledge. The four *LEPR *SNPs were *LEPR c.326A>G*, *LEPR c.668A>G*, *LEPR c.1968G>C *and *LEPR c.3024A>G*.

We did not find significant difference in the frequency of alleles, genotypes and haplotypes of the studied LEPR SNPs' in HELLP syndrome patients compared to normotensive healthy pregnant controls. Interesting finding was that in the case of LEPR *c.326A>G *(K109) the *AG *genotype was present twice more frequent in HELLP syndrome close to be statistically significant difference. The other is the frequency of the (AG AG GG AG) haplotype which was three times higher, but it was not statistically different due to the low number of cases. We need more data to get useful information on haplotypes as we had 24 in the studied population. It would be interesting to involve the fetal genotypes and haplotypes, but the present ethical regulations do not permit it.

Our data is consistent with previous research in patients who have PE, the observed allele frequencies are similar to other published results in the Hungarian population [10]. There are only a few publications on the *LEPR *gene polymorphism in PE, according to our knowledge it has not been studied in HELLP syndrome. Rigo et al. observed lower frequency of *LEPR 223AA *genotype in severely PE patients, which SNP is involved in our study also [10]. They studied the LEPR A109G and LEPR A223G using PCR-RFLP method. Based on our investigation there is no association with the LEPR SNP polymorphism and the development of HELLP syndrome. It is in agreement with previous study on PE using two LEPR SNP polymorphisms on same size of cases and controls [10]. We received similar allele and genotype frequencies on these two SNPs. The presence of the 223G allele was associated with increased insulin resistance in healthy women [32]. Insulin resistance is considered as a risk factor in PE.

Leptin receptors are widely expressed, showing that leptin has effect on several function in the body. It has an effect on angiogenesis and vascular disorders. Preliminary studies showed that platelets are the major source of leptin receptor in the circulation. This suggested that it could have effect on the development of thrombosis [33].

Our real-time PCR and melting curve analysis method is replacing the PCR-RFLP method which is widely used for SNP determinations. We can reduce the number of pipetting meanwhile reducing the risk of contamination during the analysis of the samples. The melting curve analysis makes the reliable allele determination as there are 5-10°C differences in the melting points of the alleles. The reaction is faster than the conventional PCR and we can avoid the use of separate digesting, electrophoresis and detection step which is used during PCR-RFLP. We can reach shorter detection times, less labor and favorable price.

There is a lack of information on the role of *LEPR *and its polymorphisms on the leptin levels in PE and HELLP syndrome. We determined four coding *LEPR *but we did not find correlation with the development of HELLP syndrome. Further studies are needed to reveal the molecular details of this association. We introduced the quantitative real-time PCR combined with melting curve analysis for the fast and reliable determination of the LEPR SNPs.

## Conclusion

Although certain *LEPR *haplotypes are more frequent in HELLP syndrome, we conclude that there is no compelling evidence that the four studied *LEPR *SNP polymorphisms associated with the development of HELLP syndrome.

## Competing interests

The authors declare that they have no competing interests.

## Authors' contributions

TV, LL, AM, NGT, JR and BN participated in the design of the study. TV, AM, NGT, and JR recruited the patients involved in the study. TV and BN carried out the laboratory analysis, participated in the analysis and interpretation of the data. TV and AM made the statistical analysis of the data. All the authors read and approved of the final manuscript.

## Pre-publication history

The pre-publication history for this paper can be accessed here:

http://www.biomedcentral.com/1471-2350/11/25/prepub
